# Characteristics and institutional initiatives that improve the surgical antibiotic prophylaxis use

**DOI:** 10.1186/2047-2994-4-S1-P81

**Published:** 2015-06-16

**Authors:** C Schmitt, RA Lacerda

**Affiliations:** 1 Medical-Surgical, University of São Paulo, São Paulo, Brazil

## Introduction

There is little data about adherence to surgical antibiotic prophylaxis guidelines in Brazil.

## Objectives

To recognize the institutional initiatives to improve antibiotic prophylaxis practices in neurosurgery.

## Methods

Cross-sectional observational study, carried out with a population consisting of hospitals, medical records of neurosurgical patients, Infection Control Team (ICT), surgical team. The sample of hospitals and surgical team was used for convenience and the records for each hospital was calculated based on 40% of overall adherence.

## Results

Among the nine assessed hospitals, five achieved quality certification in 2010. The mean weekly hours of ICT per hospital bed and per critical bed was 0.7 and 3.8. Eight hospitals disclosed SSI rates, seven stratified by surgical specialty, six created the antibiotic prophylaxis guidelines with the surgeons’ approval; in four the recommendations were disseminated. Of the 1,011 neurosurgeries 38 were excluded due to lack of records. Overall adherence was 10.0%. The administration route was appropriate in 100%, dose in 90.6%, indication in 90.0% and time of onset in 77.1%. There was a lower adherence regarding duration (26.1%) and a statistically significant association between hours of ICT/ICU bed (p=0.048), dissemination of surgical antibiotic prophylaxis use guidelines (p = 0.035), adherence monitoring (p = 0.024), disclosing of results (p = 0.015) and the period of the day when the surgery occurred (CI=1.7 to 6.6). Among the total of 43 anesthesiologists and surgeons interviewed more than 80% agreed with the institutional guidelines and more than 50% reported they always followed them.

## Conclusion

The number of ICT professionals/critical bed, dissemination of guidelines, monitoring and disclosing of results are associated with higher adherence regarding antibiotic prophylaxis use; period of surgery, dose (IC 1.72 - 6.65) and initial time (IC 1.12 - 3.01) and surgery type, initial time (IC 1.24 - 4.25) and duration (IC 1.09 - 2.59). The ICT had structure as required by law, but had shortcomings regarding the process of guideline implementation, monitoring and dissemination of results.

## Disclosure of interest

None declared.

